# Molecular detection of *Acanthamoeba* spp., *Naegleria fowleri* and *Vermamoeba* (*Hartmannella*) *vermiformis* as vectors for *Legionella* spp. in untreated and solar pasteurized harvested rainwater

**DOI:** 10.1186/s13071-016-1829-2

**Published:** 2016-10-10

**Authors:** Penelope H. Dobrowsky, Sehaam Khan, Thomas E. Cloete, Wesaal Khan

**Affiliations:** 1 Department of Microbiology, Faculty of Science, Stellenbosch University, Private Bag X1, Stellenbosch, 7602 South Africa; 2 Faculty of Health and Applied Sciences, Namibia University of Science and Technology, 13 Storch Street, Private Bag 13388, Windhoek, Namibia

**Keywords:** Rainwater harvesting, Solar pasteurization, *Legionella*, *Acanthamoeba*, *Vermamoeba*, *Naegleria*

## Abstract

**Background:**

*Legionella* spp. employ multiple strategies to adapt to stressful environments including the proliferation in protective biofilms and the ability to form associations with free-living amoeba (FLA). The aim of the current study was to identify *Legionella* spp., *Acanthamoeba* spp., *Vermamoeba* (*Hartmannella*) *vermiformis* and *Naegleria fowleri* that persist in a harvested rainwater and solar pasteurization treatment system.

**Methods:**

Pasteurized (45 °C, 65 °C, 68 °C, 74 °C, 84 °C and 93 °C) and unpasteurized tank water samples were screened for *Legionella* spp. and the heterotrophic plate count was enumerated. Additionally, ethidium monoazide quantitative polymerase chain reaction (EMA-qPCR) was utilized for the quantification of viable *Legionella* spp., *Acanthamoeba* spp., *V. vermiformis* and *N. fowleri* in pasteurized (68 °C, 74 °C, 84 °C and 93 °C) and unpasteurized tank water samples, respectively.

**Results:**

Of the 82 *Legionella* spp. isolated from unpasteurized tank water samples, *Legionella longbeachae* (35 %) was the most frequently isolated, followed by *Legionella norrlandica* (27 %) and *Legionella rowbothamii* (4 %). Additionally, a positive correlation was recorded between the heterotrophic plate count vs. the number of *Legionella* spp. detected (ρ = 0.710, *P* = 0.048) and the heterotrophic plate count vs. the number of *Legionella* spp. isolated (ρ = 0.779, *P* = 0.0028) from the tank water samples collected. Solar pasteurization was effective in reducing the gene copies of viable *V. vermiformis* (3-log) and *N. fowleri* (5-log) to below the lower limit of detection at temperatures of 68–93 °C and 74–93 °C, respectively. Conversely, while the gene copies of viable *Legionella* and *Acanthamoeba* were significantly reduced by 2-logs (*P* = 0.0024) and 1-log (*P* = 0.0015) overall, respectively, both organisms were still detected after pasteurization at 93 °C.

**Conclusions:**

Results from this study indicate that *Acanthamoeba* spp. primarily acts as the vector and aids in the survival of *Legionella* spp. in the solar pasteurized rainwater as both organisms were detected and were viable at high temperatures (68–93 °C).

## Background

The demand on fresh water supplies is intensifying as a result of an increase in the world’s population and urbanization, coupled with the negative effects of climate change [1–3]. Domestic rainwater harvesting systems can be utilized to augment existing surface- and groundwater supplies and in many countries it is utilized as a primary potable water source as well as for domestic and irrigation purposes. Several studies have however, highlighted that rainwater may become contaminated, especially during the harvesting process when debris, animal excreta, dust and leaves, which have accumulated on the roof catchment surface, are washed into the rainwater storage tank [4–6]. It is thus recommended that harvested rainwater is disinfected as numerous pathogens, including those that are opportunistic in nature, have previously been detected in this water source and are of a human health concern [7–12].

Dobrowsky et al. [13], indicated that a closed-coupled solar pasteurization system operating at temperatures greater than 72 °C can be utilized to treat harvested rainwater as the level of heterotrophic bacteria, *Escherichia coli* (*E. coli*) and total coliforms were reduced to below the detection limit and were within the respective drinking water guidelines [14–16]. However, PCR assays confirmed the presence of *Yersinia* spp., *Legionella* spp., and *Pseudomonas* spp., at temperatures greater than 72 °C, with *Legionella* spp. persisting at temperatures greater than 90 °C. A follow-up study performed by Reyneke et al. [17], indicated that *Legionella* spp. may be entering a viable but non-culturable (VBNC) state as intact *Legionella* cells were detected at temperatures of up to 95 °C using ethidium monoazide (EMA) quantitative PCR (EMA-qPCR).

*Legionella* spp. exhibit a number of mechanisms enabling them to withstand environmental stresses such as heat treatment. These include associations with at least 20 protozoan hosts including *Acanthamoeba* spp., *Naegleria* spp., *Vermamoeba* (*Hartmannella*) *vermiformis* and *Vahlkampfia* spp. and two species of ciliated protozoa, including *Tetrahymena* spp. and *Cyclidium* spp. [18]. Their association with free-living amoeba (FLA) is especially effective as the amoeba host provides nutrients including, amino acids for the proliferation of *Legionella* spp. and a protective environment when *Legionella* spp. are enclosed in the cysts of the amoeba species [19, 20].

Of the genera belonging to the FLA, *Acanthamoeba* spp., *Naegleria fowleri* and *V. vermiformis* are the most frequently isolated from water samples [21–23], including samples from hot water systems [19, 24, 25]. Moreover, *Acanthamoeba* spp. and *N. fowleri* are associated with human and animal infections, including amoebic keratitis and severe brain pathologies [26–29]. The life-cycles of these FLAs are then divided into two stages. First, in the form of a vegetative trophozoite, the organism is able to feed and replicate. Secondly, a cyst is formed under unfavourable environmental conditions and this allows the organism to withstand nutrient starvation, heat, cold, desiccation and biocidal treatments [29–31]. Although there is limited data regarding FLA resistance to various disinfection procedures, they are a potential risk to public health not only because of the transmission of the protozoa themselves, but because they harbour a range of microbial pathogens including *Legionella* spp., *Listeria monocytogenes*, *Pseudomonas aeruginosa* and *Mycobacterium* spp., amongst other species [29, 32, 33].

Generally during the process of phagocytosis, the amoeba will engulf avirulent bacterial cells and form a phagosome. The phagosome then fuses with the lysosome, containing lysozymes, which degrade the bacterial cells [34]. Although there are differences in host-cell trafficking processes amongst *L. pneumophila*, *L. micdadei* and *L. longbeachae*, virulent *Legionella* spp. have the ability to halt the phagosome-lysosomal degradation pathway of the amoeba. This implies that the phagosome (containing the *Legionella*) does not undergo sequential maturation and therefore does not ultimately fuse with degradative lysosomes [35, 36]. The *Legionella* instead form a *Legionella* containing vacuole by recruiting secretory vesicles from the endoplasmic reticulum exit sites and mitochondria of the amoeba to the plasma membrane of the *Legionella* containing vacuole [36–38]. Owing to the proteins of the Type IVB defect in organelle trafficking/intracellular multiplication (Dot/Icm) secretion system, that aid in the establishment and the preservation of the *Legionella* containing vacuole, *Legionella* are then able to proliferate in this protective rough endoplasmic reticulum-like compartment [38, 39]. Once nutrients within the *Legionella* containing vacuole become limiting, *Legionella* will kill the amoeba and escape, where after they either establish a new replicative niche within a new host or continue to survive as planktonic cells and/or within biofilms as sessile cells [39, 40]. It has then been suggested that the growth of *Legionella* within amoeba hosts in the environment, is required to select or maintain virulent strains of *Legionella* able to cause Legionnaires’ disease [18, 39].

Numerous *Legionella* spp. (e.g. *L. pneumophila* and *L. longbeachea*, amongst others) have been known to cause the acute potentially fatal form of pneumonia as part of a multisystem disease known as Legionnaires’ disease (also referred to as Legionellosis or Legion Fever) [41] or a milder form of pulmonary infection known as Pontiac fever, which is a flu like illness [42]. Since the outbreak of Legionnaires’ disease has previously been linked to roof-harvested rainwater systems [8, 43] and hot water distribution systems [44], the aim of the current study was to isolate and identify the primary *Legionella* spp. contaminating a harvested rainwater and a solar pasteurization (SOPAS) system (used for the treatment of roof-harvested rainwater) and to identify possible vectors including *Acanthamoeba* spp., *V. vermiformis* and *N. fowleri*, enabling their resistance and persistence. The viability of the FLA’s as well as *Legionella* spp. at temperatures greater than 68 °C was also determined using EMA-qPCR. The enumeration of heterotrophic bacteria was included as previous studies have suggested that a correlation may exist between the heterotrophic plate count and *Legionella*, as *Legionella* multiply in biofilms as a survival strategy in the environment [45–47]. Additionally, the heterotrophic plate count was utilized to monitor the change in the number of viable heterotrophic bacteria before and after pasteurization [48]. Unpasteurized and pasteurized rainwater samples treated at temperatures of 45 °C, 65 °C, 68 °C, 74 °C, 84 °C and 93 °C, were collected for the enumeration of the heterotrophic plate count and the isolation of *Legionella* spp. However, as previous research has indicated that the indicator counts are reduced to below the detection limit at temperatures greater than 70 °C [13, 17], the samples collected at temperatures of 68 °C, 74 °C, 84 °C and 93 °C only were utilized for the EMA-qPCR experiments.

## Methods

### Sample site and collection

The Apollo™ solar pasteurization system previously described by Dobrowsky et al. [13] was utilized for the treatment of harvested rainwater stored in a polyethylene rainwater harvesting (RWH) tank (2000 l). The RWH tank and pasteurization system were installed at the Welgevallen experimental farm (33°56′36.19″S, 18°52′6.08″E), Stellenbosch University, Western Cape, South Africa, during July 2013. Samples (5 l) pasteurized at 45 °C, 65 °C, 68 °C, 74 °C, 84 °C and 93 °C were collected from the Apollo™ solar pasteurization system during September and October 2015, with six corresponding unpasteurized tank water samples (5 l) collected from the connecting RWH tank. The pH and temperature of the tank water samples were recorded at the sampling site, using a using a handheld pH55 pH/temperature meter (Martini Instruments, North Carolina, USA) and an alcohol thermometer, respectively.

Ambient temperature for the Stellenbosch area during 2015 were obtained from the South African Weather Services (Pretoria, South Africa), while global horizontal irradiance (GHI; W/m^2^) data were obtained from Stellenbosch Weather Services, Engineering Faculty, Stellenbosch University (http://weather.sun.ac.za/).

### Enumeration of the heterotrophic plate count

For the enumeration of the heterotrophic plate count, a serial dilution (1:10) was prepared for each unpasteurized and pasteurized tank water sample, respectively, and 100 μl of each undiluted and diluted (10^-1^–10^-2^) sample was spread plated onto Reasoner’s 2A agar (R2A agar; Difco Laboratories, Detroit, Michigan, USA), with the plates incubated at 37 °C for up to 4 days in accordance with Standard Methods 9215 C, American Public Health Association [49].

### Isolation of *Legionella* spp. from pasteurized and unpasteurized tank water samples

*Legionella* spp. were recovered from the pasteurized (45 °C, 65 °C, 68 °C, 74 °C, 84 °C and 93 °C) and unpasteurized tank water samples according to the procedure outlined by the Centers for Disease Control and Prevention [50]. Briefly, 500 ml of each sample collected from the domestic rainwater harvesting tank and from the pasteurization system, respectively, was filtered through a sterile GN-6 Metricel® S-Pack Membrane Disc Filter (Pall Life Sciences, Michigan, USA) with a pore size of 0.45 μm and a diameter of 47 mm. The filtration flow rate was approximately ≥ 65 ml/min/cm^2^ at 0.7 bar (70 kPa). The filters were then aseptically removed from the filtration system and were placed into sterile 50 ml centrifuge tubes containing sterile water (5 ml). If more than one filter was required, additional filters were added to the same sample tube. The centrifuge tubes were then vortexed to detach the cells from the filters. As *Legionella* detection and isolation may be hampered by the growth of non-*Legionella* background flora [51], the selective detection for the unpasteurized tank water samples was increased by pre-incubating the cell suspension at 50 °C for 30 min before cultivation [50]. Thereafter, 100 μl of the cell suspension was spread plated onto buffered charcoal yeast extract (BCYE) agar containing ACES [*N*-(2-acetamido)-2-aminoethanesulfonic acid] buffer/potassium hydroxide (1.0 g/l), ferric pyrophosphate (0.025 g/l), alpha-ketoglutarate (0.10 g/l) and *L*-cysteine HCL (0.04 g/l) (Oxoid, Hampshire, England), BCYE agar supplemented with glycine, vancomycin, polymyxin B and cycloheximide (GVPC) and charcoal yeast extract (CYE) agar base without supplements (Oxoid, Hampshire, England).

All plates were then incubated at 35 °C for approximately 10 days. Colonies exhibiting a convex and round with entire morphology that appeared on the BCYE and GVBC media and not on the CYE medium were selected for further analysis. To confirm the presence of presumptive *Legionella* spp., colonies were streaked onto BCYE agar and Nutrient Agar (NA; Merck, Gauteng, South Africa) as a preliminary identification strategy. Colonies that grew only on BCYE agar and not NA were presumptively classified as *Legionella* spp. and were utilized for further analysis.

### Total genomic DNA extractions from presumptive *Legionella* isolates

Total genomic DNA (gDNA) extractions were performed for presumptive *Legionella* spp. isolated from the tank water samples present on the BCYE and GVBC media and not on the CYE media. Before the extraction of DNA, presumptive *Legionella* colonies were inoculated into buffered yeast extract (BYE) broth supplemented with ACES buffer/potassium hydroxide (1.0 g/l), ferric pyrophosphate (0.025 g/l), alpha-ketoglutarate (0.10 g/l) and *L*-cysteine HCL (0.04 g/l) according to Edelstein & Edelstein [52]. Presumptive *Legionella* cultures were then incubated at ± 35 °C for 4 days. Total gDNA was extracted from the cultures using the boiling method previously described by Ndlovu et al. [53].

### Ethidium monoazide (EMA) treatment and total gDNA extractions from pasteurized and unpasteurized tank water samples

For the detection and quantification of *Legionella* spp., *Acanthamoeba* spp., *N. fowleri* and *V. vermiformis* total gDNA was extracted from pasteurized (68 °C, 74 °C, 84 °C, 93 °C) and the corresponding unpasteurized tank water samples. For this, 1 l of each sample was first subjected to flocculation as previously described by Dobrowsky et al. [54]. Briefly, 2 ml of CaCl_2_ (1 M) and 2 ml of Na_2_HPO_4_ (1 M) were added to each sample before the samples were stirred (room temperature) for 5 min. The samples were then concentrated by filtration as outlined above. For the detachment of cells from the filters, the filters were transferred to 4 ml citrate buffer (0.3 M, pH 3.5) and were vortexed. The filters were subsequently removed and the 4 ml suspension was centrifuged at 16 000× *g* for 10 min. After the removal of the supernatant the pellet was re-suspended in 1 ml sterile MilliQ water before EMA treatment.

Ethidium monoazide (2.5 μg/ml) was added to the 1 ml of concentrated sample according to Delgado-Viscogliosi et al. [55] and Chang et al. [56]. The suspension was then vortexed vigorously and placed on ice for 10 min in the dark. To cross link the EMA to the naked DNA, the samples were kept horizontal on ice and were exposed to a 500 W halogen light for 15 min at a distance of 20 cm. Following centrifugation (16 000× *g*, 5 min), the supernatant was removed and the pellet was washed with 1 mL NaCl (0.85 %). The sample was centrifuged (16 000× *g*, 5 min) and the pellet was re-suspended in 700 μl lysis buffer. The extraction of total gDNA was then performed using the Soil Microbe DNA MiniPrep™ Kit (Zymo Research, Irvine, USA) according to manufacturer’s instructions.

### Conventional PCR assays for the identification of *Legionella* isolates

For the identification of the presumptive *Legionella* isolates, DNA was extracted from each isolate as outlined above. The primer set LEG 225/LEG 858 was then utilized to amplify 634 bp of the 16S rRNA sequence as previously described by Miyamoto et al. [57] (Table 1). The PCR mix consisted of 10 μl of 5× Green GoTaq® Flexi Buffer (1×; Promega, Madison, USA), 4 μl MgCl_2_ (2.0 mM; Promega), 0.5 μl of each dNTP (0.1 mM; Thermo Fischer Scientific, Waltham, USA), 2 μl of each PCR primer (LEG 225 and LEG 858; 0.4 μM), 0.3 μl of GoTaq® Flexi DNA Polymerase (1.5U; Promega) and 2 μl of template DNA. All conventional PCR mixtures consisted of a final volume of 50 μl. The PCR cycling parameters were as follows: initial denaturation at 95 °C (1.5 min) followed by 30 cycles of denaturation at 94 °C (10 s), annealing at 64 °C (1 min) and elongation at 74 °C (1 min). A final extension was included at 72 °C (10 min).

**Table 1:** Primers and amplification conditions utilized in the current study for the identification and quantification of *Legionella* spp., *Acanthamoeba* spp., *Naegleria fowleri* and *Vermamoeba* (*Hartmannella*) *vermiformis* in pasteurized and unpasteurized tank water samples

Organism	Primer name	Primer sequence (5'–3')	Gene (size, bp)	Amplification conditions	Reference
*Legionella* spp. (Identification)	LEG 225	AAGATTAGCCTGCGTCCGAT	16S rRNA (634)	95 °C (1.5 min) followed by 30 cycles of 94 °C (10 s), 64 °C (1 min) and 74 °C (1 min). Final extension: 72 °C (10 min).	Miyamoto et al. [57]
	LEG 858	GTCAACTTATCGCGTTTGCT			
*Legionella* spp*.*	Leg F	CTAATTGGCTGATTGTCTTGAC	23S-5S rRNA (259)	95 °C (1 min) followed by 45 cycles of 95 °C (15 s), 60 °C (15 s) and 72 °C (11 s)	Herpers et al. [58]
	Leg R	CAATCGGAGTTCTTCGTG			
*Acanthamoeba* spp.	AcantF900	CCCAGATCGTTTACCGTGAA	18S rDNA (±180)	95 °C (1 min) followed by 45 cycles of 95 °C (15 s), 60 °C (1 min) and 72 °C (40 s)	Qvarnstrom et al. [59]
	AcantR1100	TAAATATTAATGCCCCCAACTATCC			
*Naegleria fowleri*	NaeglF192	GTGCTGAAACCTAGCTATTGTAACTCAGT	18S rDNA (153)	95 °C (1 min) followed by 45 cycles of 95 °C (15 s), 64 °C (1 min) and 72 °C (1 min)	Qvarnstrom et al. [59]
	NaeglR344	CACTAGAAAAAGCAAACCTGAAAGG			
*Vermamoeba* (*Hartmannella*) *vermiformis*	Hv1227F	TTACGAGGTCAGGACACTGT	18S rRNA (502)	95 °C (3 min) followed by 45 cycles of 95 °C (20 s), 58 °C (30 s) and 72 °C (40 s)	Kuiper et al. [60]
	Hv1728R	GACCATCCGGAGTTCTCG			

### Quantification of viable *Legionella* spp., *Acanthamoeba* spp., *N. fowleri* and *V. vermiformis* in pasteurized and unpasteurized tank water samples

For the quantification of viable *Legionella* spp., *Acanthamoeba* spp., *N. fowleri* and *V. vermiformis* in pasteurized (68 °C, 74 °C, 84 °C, 93 °C) and unpasteurized tank water samples, quantitative PCR (qPCR) was performed using a LightCycler ® 96 (Roche, Gauteng, South Africa) following EMA treatment. For all qPCR assays, to a final reaction volume of 20 μl, using the FastStart Essential DNA Green Master Mix (Roche Applied Science, Mannheim, Germany), the following were added: 10 μl FastStart Essential DNA Green Master Mix (2×), 5 μl template DNA, and 0.4 μl of each primer (0.2 μM).

For the quantification of *Legionella* spp. in pasteurized and unpasteurized tank water samples, the primers and qPCR parameters according to Herpers et al. [58] were utilized (Table 1). To generate a standard curve for the quantification of *Legionella* spp., the purified conventional PCR product obtained by amplifying the 256 bp product from *L. pneumophila* ATCC 33152 was utilized.

For the quantification of *Acanthamoeba* spp. in pasteurized and unpasteurized tank water samples, the primers and qPCR parameters as previously described by Qvarnstrom et al. [59] were utilized (Table 1). To generate the standard curve for the quantification of *Acanthamoeba* spp., the 180 bp PCR product amplified from gDNA of *A. mauritaniensis* ATCC 50677 was cloned into the pGEM T-easy vector system (Promega Corp.) according to the manufacturer’s instructions. Once the plasmid had been sequenced, the plasmid containing the correct insert was used to generate the standard curve.

Additionally, for the quantification of *N. fowleri* in pasteurized and unpasteurized tank water samples, the primers and qPCR parameters as outlined by Qvarnstrom et al. [59] were utilized (Table 1). To generate the standard curve for the quantification of *N. fowleri* a purified 153 bp PCR product obtained by screening a 1 l tank water sample from a domestic rainwater harvesting tank located at Stellenbosch University (GPS coordinates: 33°55'51.1"S, 18°51'56.7"E) using the NaeglF192/NaeglR344 primer set was cloned into the pGEM T-easy vector system (Promega Corp.) according to the manufacturer’s instructions. Once the plasmid had been sequenced, the plasmid containing the correct insert was used to generate the standard curve for the quantification of *N. fowleri*.

For the quantification of *V. vermiformis* in pasteurized and unpasteurized tank water samples, the primers and qPCR parameters according to Kuiper et al. [60] were utilized (Table 1). To generate a standard curve for the quantification of *V. vermiformis*, the purified conventional PCR product (502 bp) obtained by screening a 1 l tank water sample from a domestic rainwater harvesting tank located at Stellenbosch University (GPS coordinates: 33°55'51.1"S, 18°51'56.7"E) using the Hv1227F/Hv1728R primer set was utilized.

The concentration of the purified PCR products (*Legionella* spp. and *V. vermiformis*) and plasmid DNA (*Acanthamoeba spp.* and *N. fowleri*) were quantified using the NanoDrop® ND-1000 (Nanodrop Technologies Inc., Wilmington, Delaware, USA) in triplicate at CAF. Serial 10-fold dilutions (10^9^ to 10^1^) of the sequenced conventional PCR products and plasmid DNA were prepared in order to generate the standard curves for each respective organism. A concentration of 1.00 × 10^9^ gene copies/μl was prepared for the dilution with the highest copy number and a concentration of 1.00 × 10^1^ gene copies/μl was prepared for the dilution with the lowest copy number. Standard curves generated by plotting quantitative cycle (Cq) values *vs* the log concentrations of standard DNA as previously described by Chen and Chang [61], were then used to determine the number of gene copies of each of the organisms. Melt curve analysis was included for all SYBR green real-time PCR assays in order to verify specificity of the primer set by ramping the temperature from 65 to 97 °C at a rate of 0.2 °C/s with continuous fluorescent signal acquisition at 5 readings/°C.

### Sequencing of PCR amplicons

The PCR amplicons of each presumptive *Legionella* isolate, the PCR products used as positive controls to generate the standard curves for each qPCR assay and representative products of each of the qPCR assays of each organism were then purified using the DNA Clean & Concentrator™-5 Kit (Zymo Research) and were sent for sequencing at the CAF, Stellenbosch University. Chromatograms of each sequence were examined as outlined in Dobrowsky et al. [12] and all sequences were submitted as a query to BLAST for a sequence similarity search against the NCBI databases (https://blast.ncbi.nlm.nih.gov/Blast.cgi).

### Inter- and intra-assay reproducibility and the lower limit of detection

To establish the inter-assay reproducibility of the qPCR assays optimized for each respective organism, the coefficient of variation (CV) was determined using the concentrations of nine dilutions (10^9^ to 10^1^) of conventional PCR products and plasmid DNA that were quantified in duplicate during three separate qPCR experiments. In addition, the CV for intra- assay repeatability was calculated using the concentrations of nine dilutions (10^9^ to 10^1^) for each qPCR assay [62–65]. In order to eliminate any PCR inhibitors, samples resulting in end-point fluorescence (EPF) values of less than 3.15 were diluted (10×) and the qPCR experiment was repeated for these samples. The minimum number of gene copies (highest dilution) that could be measured accurately within an assay was considered the lower limit of detection for each organism [63].

### Statistical analysis

The data obtained from the microbial and physical analysis of the tank water samples collected, were assessed using the Statistical software package, Statistica™ version 13.0 (Statsoft Inc.). Before the data analysis, the Shapiro-Wilk test was used to test the normality of data sets. The gene copies of *Legionella* spp., *Acanthamoeba* spp., *V. vermiformis* and *N. fowleri* obtained for the pasteurized and unpasteurized tank water samples were assessed for nonparametric differences using the Mann-Whitney U test. Thus, the temperature of the tank water samples, before and after pasteurization was used as a single, ordinal variable. Spearman’s rank (ρ) correlation tests were performed to establish correlations between different microbiological (the heterotrophic plate count, number of *Legionella* isolates, the gene copies of *Legionella* spp., *Acanthamoeba* spp., *V. vermiformis* and *N. fowleri* obtained before and after pasteurization) and physical parameters (pH and temperature of tank water samples) as previously described by Wang et al. [66]. Significance was set at a *P*-value of ≤ 0.05 for all statistical analyses performed.

## Results

### Physical parameters of pasteurized and unpasteurized tank water samples collected during the sampling period (September–October 2015)

Pasteurized (45 °C, 65 °C, 68 °C, 74 °C, 84 °C and 93 °C) tank water samples were collected from the Apollo™ solar pasteurization system with corresponding unpasteurized tank water samples collected from the RWH tank during September and October 2015. The average daily ambient temperature ranged from 15.7 °C (September 2015) to 18.5 °C (October 2015). Additionally, as the Apollo™ solar pasteurization system relies on radiation from the sun to heat the tank water, the average total GHI was recorded at 8288.9 W/m^2^ during September 2015 and 11574.6 W/m^2^ during October 2015. The temperature of the water samples collected from the RWH tank ranged from the lowest temperature of 18 °C (15.09.2015) to the highest recorded temperature of 31 °C (27.10.2015). An average pH of 8.0 (range: 7.9–8.1) was recorded for unpasteurized tank water samples which then increased to pH 8.3 (range: 8.2–8.5) after pasteurization (Table 2).

**Table 2:** Microbiological parameters and physical parameters determined for pasteurized and unpasteurized harvested rainwater samples

Date	Unpasteurized and pasteurized sample temp. (°C)	pH	Heterotrophic plate count (CFU/ml)	No. of *Legionella* isolates obtained	Gene copies/ml			
					*Legionella* spp.	*Acanthamoeba* spp.	*Vermamoeba vermiformis*	*Naegleria fowleri*
22.10.2015	24	8.4	1.5 × 10^6^	9	6.5 × 10^4^	9.8 × 10^4^	5.7 × 10^6^	1.6 × 10^5^
	68^a^	8.4	BDL^b^	BDL^b^	3.2 × 10^3^	8.4 × 10^3^	9.4 × 10^3^	LLOD^d^
22.10.2015	25	8.1	1.0 × 10^6^	2	4.5 × 10^4^	8.3 × 10^4^	3.9 × 10^4^	6.4 × 10^4^
	74^a^	8.5	BDL^b^	BDL^b^	9.2 × 10^3^	5.2 × 10^3^	LLOD^c^	LLOD^d^
19.10.2015	21	8.3	6.8 × 10^5^	1	5.7 × 10^6^	1.3 × 10^5^	1.4 × 10^5^	9.2 × 10^4^
	84^a^	8.2	BDL^b^	BDL^b^	2.3 × 10^3^	1.7 × 10^4^	LLOD^c^	LLOD^d^
27.10.2015	31	8.4	2.7 × 10^5^	3	8.2 × 10^6^	6.5 × 10^4^	3.2 × 10^5^	1.0 × 10^6^
	93^a^	8.2	BDL^b^	BDL^b^	1.1 × 10^3^	1.4 × 10^4^	LLOD^c^	LLOD^d^

^a^Pasteurized rainwater sample

^b^ *BDL* below detection limit

^c^ *LLOD* lower limit of detection: *Vermamoeba vermiformis* (< 5–8 gene copies/μl)

^d^ *LLOD* lower limit of detection: *Naegleria fowleri* (< 12–17 gene copies/μl)

### The heterotrophic plate count and culturing of *Legionella* spp

For all unpasteurized tank water samples (*n* = 6), the heterotrophic plate count numbers ranged from 2.7 × 10^5^ CFU/ml to 1.5 × 10^6^ CFU/ml and were above the Department of Water Affairs and Forestry (DWAF) [14] guideline of 100 CFU/ml (Table 2). Additionally, the heterotrophic plate count were above the DWAF [14] guidelines following pasteurization at 45 °C (1.5 × 10^5^ CFU/ml) and 65 °C (4.7 × 10^2^ CFU/ml), respectively (results not shown). However, after the pasteurization treatment for the temperatures ranging from 68 to 93 °C, heterotrophic plate counts were reduced to below the detection limit (< 1 CFU/ml) and were within the DWAF guidelines (Table 2).

### Conventional PCR for the identification of *Legionella* isolates

**Fig. 1:**
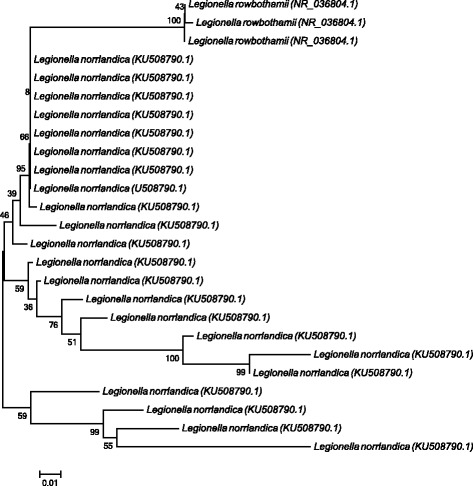
Phylogenetic tree constructed from sequences of PCR products of *Legionella norrlandica* and *Legionella rowbothamii* isolates by means of the maximum composite likelihood function (evolutionary history) and neighbor-joining method using the program MEGA 5. The numbers found adjacent to the nodes represent the data (percentages) from 1,000 exploratory bootstrap trials. Bar: 0.002 changes per site. The *L. norrlandica* and *L. rowbothamii* isolates were obtained from unpasteurized tank water samples

Culture based methods were then utilized to isolate *Legionella* spp. from all pasteurized (*n* = 6) and unpasteurized (*n* = 6) tank water samples. While no *Legionella* spp. were isolated from pasteurized tank water samples (45 °C to 93 °C), *Legionella* spp. were isolated from all the unpasteurized tank water samples utilizing culturing methods (Table 2). A total of 82 *Legionella* isolates were obtained overall from all the unpasteurized samples and all the resulting DNA sequences of the *Legionella* isolates displayed similarities to sequences of *Legionella* spp. recorded on NCBI. *Legionella longbeachae* (*n* = 29; GenBank accession no: FN650140.1, JN606078.1, NR_102800.1) was the species most frequently isolated from the unpasteurized tank water samples (results not shown), followed by *Legionella norrlandica* (*n* = 22) and *Legionella rowbothamii* (*n* = 3) (Fig. 1; accession numbers included), the remaining 28 isolates were undetermined *Legionella* species (results not shown) and BLAST analysis indicated the presence of uncultured *Legionella* spp. (GenBank accession no: HQ111985.1, HQ111937.1, GU185995.1) and *Legionella* spp. (GenBank accession no: JN380993.1, JN380988.1).

### Quantification of viable *Legionella* spp. present in pasteurized and unpasteurized tank water samples

As the heterotrophic plate count was reduced to below the detection limit following pasteurization at 68 °C to 93 °C, viable *Legionella* spp. were quantified in samples pasteurized at 68 °C, 74 °C, 84 °C, 93 °C and the four corresponding unpasteurized tank water samples (Table 2). For all unpasteurized tank water samples, the concentration of viable *Legionella* ranged from 4.5 × 10^4^ gene copies/ml (25 °C) to 8.2 × 10^6^ gene copies/ml (31 °C) (Table 2). After pasteurization treatment, the highest concentration of *Legionella* was detected at 74 °C (9.2 × 10^3^ gene copies/ml) which then decreased to 1.1 × 10^3^ gene copies/ml following pasteurization at 93 °C as indicated in Table 2. The number of *Legionella* gene copies then decreased by 1-log (87.2 %) following pasteurization at 68 °C and 74 °C, respectively. In contrast, a 3-log reduction (> 99.9 %) in *Legionella* gene copies was observed following pasteurization at 84 °C, while a 4-log (> 99.9 %) reduction in *Legionella* gene copies was observed following pasteurization at 93 °C. Overall, the number of viable *Legionella* gene copies decreased significantly (Z = 3.034; *P* = 0.0024) by an average of 2-logs (93.6 %) following pasteurization at 68–93 °C. Representative qPCR products were sequenced and *Legionella anisa* (GenBank accession no: JN001853.1, Z24682.1) and *Legionella monrovica* (GenBank accession no: Z24729.1) were detected in representative pasteurized and unpasteurized tank water samples following BLAST analysis, respectively.

### Quantification PCR efficiency, reproducibility and lower limit of detection

A linear range of quantification from 10^9^ to 10^1^ gene copies per μl of DNA extracts was observed for all standard curves produced for the quantification of *Legionella* spp., *Acanthamoeba* spp., *V. vermiformis* and *N. fowleri*, respectively. As indicated in Table 3, the qPCR assays had amplification efficiencies that ranged from 1.86 to 1.94 (*Legionella* spp.), 1.92 to 1.95 (*Acanthamoeba* spp.), 1.85 to 1.89 (*V. vermiformis)* and 1.90 to 2.04 (*N. fowleri*). The optimum amplification efficiency is measured at 2.00 and corresponds to a doubling of copy number for every PCR cycle [67]. The correlation coefficient (*r* ^2^) ranged from 0.99 to 1.00 for all qPCR assays performed for *Legionella* spp., *Acanthamoeba* spp. and *N. fowleri* and 0.98 to 1.00 for all qPCR assays performed *V. vermiformis*, respectively (Table 3). The qPCR lower limit of detection was recorded at 8–12 gene copies/μl for *Legionella* spp., 2–6 gene copies/μl for *Acanthamoeba* spp., 5–8 gene copies/μl for *V. vermiformis* and 12–17 gene copies/μl for *N. fowleri* (Table 3). The qPCR assays demonstrated good reproducibility as the mean inter-and intra- assay coefficient of variation (CV) values and standard deviations (SD) were less than 1 and 5 % for all qPCR assays, respectively (Table 3).

**Table 3:** The lower limit of detection (LLOD), amplification efficiency, correlation coefficient (*r* ^*2*^), intra- and inter-assay reducibility within the range of 10^9^ to 10^1^ gene copies/μl of each qPCR assay

Organisms assayed	LLOD (gene copies/μl)	Amplification efficiency (%)	Correlation coefficient (*r* ^*2*^)	Mean ± SD of CV	
				Intra-assay	Inter-assay
*Legionella* spp.	8–12	1.86 (93)–1.94 (97)	0.99–1.00	0.160 ± 0.257	0.251 ± 0.220
*Acanthamoeba* spp.	5–11	1.92 (96)–1.95 (98)	0.99–1.00	0.142 ± 0.283	0.192 ± 0.225
*Vermamoeba vermiformis*	5–8	1.85 (93)–1.89 (95)	0.98–1.00	0.09 ± 0.112	0.129 ± 0.092
*Naegleria fowleri*	12–17	1.90 (95)–2.04 (102)	0.99–1.00	0.195 ± 0.410	0.348 ± 0.251

*Abbreviations: CV* coefficient of variation, *LLOD* lower limit of detection, *SD* standard deviation

### Quantification of FLAs present in pasteurized and unpasteurized tank water samples

Following EMA treatment and DNA extractions, qPCR was performed for the quantification of viable *Acanthamoeba* spp., *V. vermiformis* and *N. fowleri* in samples pasteurized at 68 °C, 74 °C, 84 °C, 93 °C and the corresponding unpasteurized tank water samples, respectively (Table 2). As indicated in Table 2, gene copies of *Acanthamoeba* ranged from 6.5 × 10^4^ gene copies/ml (31 °C) to 1.3 × 10^5^ gene copies/ml (21 °C) for all unpasteurized tank water samples. After pasteurization treatment, the gene copies of *Acanthamoeba* spp. decreased and ranged from 5.2 × 10^3^ gene copies/ml (74 °C) to 1.7 × 10^4^ gene copies/ml (84 °C) and 1.4 × 10^4^ gene copies/ml (93 °C) (Table 2). Overall, the number of *Acanthamoeba* gene copies decreased significantly (*Z* = -3.183; *P* = 0.0015) by 1-log (87.3 %) following pasteurization at 68–93 °C. *Acanthamoeba* genotype T4 (GenBank accession no: KT892923.1), genotype T15 (GenBank accession no: KT892848.1) and *Acanthamoeba lenticulata* (GenBank accession no: KX018047.1) were detected in representative pasteurized and unpasteurized tank water samples, respectively.

For all unpasteurized tank water samples, the gene copies of viable *V. vermiformis* ranged from 3.9 × 10^4^ gene copies/ml (25 °C) to 5.7 × 10^6^ gene copies/ml (24 °C) (Table 2). Following pasteurization, the gene copies of *V. vermiformis* decreased and ranged from 9.4 × 10^3^ gene copies/mL (68 °C) to below the lower limit of detection (< 5–8 gene copies/μl) for the remainder of the pasteurized samples (74 °C to 93 °C). A 3-log reduction (99.9 %) in *V. vermiformis* gene copies was observed following pasteurization at 68 °C while a 5-log reduction (> 99.9 %) in gene copies of viable *V. vermiformis* was observed following pasteurization at 74 °C to 93 °C, respectively. Overall, the number of gene copies of *V. vermiformis* (GenBank accession no: KT185625.1) decreased significantly (*Z* = 3.067; *P* = 0.0021) by 5-log (> 99.9 %) following pasteurization at 68–93 °C.

For all unpasteurized tank water samples, gene copies of viable *N. fowleri* ranged from 6.4 × 10^4^ gene copies/ml (25 °C) to 1.0 × 10^6^ gene copies/ml (31 °C) (Table 2). Following pasteurization, the gene copies of *N. fowleri* decreased to below the lower limit of detection (< 12–17 gene copies/μl) for all tank water samples pasteurized at 68 °C, 74 °C, 84 °C and 93 °C, respectively (Table 2). A 5-log reduction (> 99.9 %) in *N. fowleri* gene copies was observed following pasteurization at 68 °C to 84 °C, respectively, while a 6-log reduction (> 99.9 %) in gene copies of *N. fowleri* was observed following pasteurization at 93 °C. Overall, the number of gene copies of viable *N. fowleri* (GenBank accession no: JQ271702.1, JQ271704.1) decreased significantly (*Z* = 3.308; *P* = 0.001) by 5.2-log (> 99.9 %) following pasteurization at 68–93 °C.

### Associations of microbiological parameters and abiotic factors

As indicated in Table 4, Spearman’s (ρ) correlations were noted between parameters measured throughout the study. For example, positive correlations were observed between the heterotrophic plate count and the number of *Legionella* isolates obtained (ρ = 0.779, *P* = 0.0028) and the gene copies of *Legionella* spp. (ρ = 0.710, *P* = 0.048), *Acanthamoeba* spp. (ρ = 0.862, *P* = 0.006), *V. vermiformis* (ρ = 0.858, *P* = 0.006) and *N. fowleri* (ρ = 0.810, *P* = 0.015), respectively. Additionally, significant positive correlations were observed between the number of *Legionella* isolates obtained *vs* the gene copies of *Legionella* spp. (ρ = 0.812, *P* = 0.0014), *Acanthamoeba* spp. (ρ = 0.761, *P* = 0.028), *V. vermiformis* (ρ = 0.936, *P* = 0.001) and *N. fowleri* (ρ = 0.946, *P* = 0.0004), respectively. The number of *Legionella* spp. gene copies/ml were also positively correlated to the gene copies of the amoeba detected including *V. vermiformis* (ρ = 0.854, *P* = 0.001), *N. fowleri* (ρ = 0.913, *P* = 0.002) and *Acanthamoeba* spp. (ρ = 0.643, *P* = 0.085). Moderate to high correlations were then detected between the number of *Acanthamoeba* spp. gene copies *vs* the number of *V. vermiformis* (ρ = 0.756, *P* = 0.03) and *N. fowleri* gene copies (ρ = 0.845, *P* = 0.028), respectively. A high correlation was also established between the gene copies of *V. vermiformis* and of *N. fowleri* (ρ = 0.936, *P* = 0.001).

**Table 4:** Spearman rank order correlation coefficients (ρ) of parameters investigated in this study

Parameter	Temperature (°C)	pH	HPC/ml	No. of isolates (Culture)	*Legionella* spp.	*Acanthamoeba* spp.	*Vermamoeba vermiformis*
Temperature (°C)	–						
pH	0.367	–					
HPC/ml	-0.847**	-0.246	–				
No. of isolates (Culture)	-0.885**	-0.374	0.779**	–			
*Legionella* spp.	-0.833**	0.306	0.710*	0.812*	–		
*Acanthamoeba* spp.	-0.809*	-0.356	0.862**	0.761*	0.643	–	
*Vermamoeba vermiformis*	-0.854**	0.176	0.858**	0.936**	0.854**	0.756*	–
*Naegleria fowleri*	-0.761**	0.118	0.810*	0.946**	0.913**	0.761*	0.936**

*P < 0.05, **P < 0.01

## Discussion

The heterotrophic plate count for all unpasteurized and tank water samples pasteurized at 45 °C and 65 °C were above the DWAF guideline for drinking water, with the heterotrophic plate count reduced to below the detection limit (< 1 CFU/ml) following pasteurization at 68 °C to 93 °C. The heterotrophic plate count represents only the culturable portion of the general bacterial community that are present in a water source and results indicated that pasteurization treatment at temperatures above 68 °C are effective as heterotrophic plate count counts in the treated samples corresponded to drinking water guidelines [14]. Previously, Sommer et al. [68] indicated that faecal coliforms were inactivated in river water at temperatures above 70 °C utilizing solar pasteurization. Moreover, Dobrowsky et al. [13] indicated that a closed-coupled solar pasteurization system operating at temperatures above 72 °C reduced the level of the heterotrophic plate count, *E. coli* and total coliforms to below the detection limit in harvested rainwater. In the current study a positive correlation between the heterotrophic plate count and the number of culturable *Legionella* present and the gene copies of viable *Legionella* spp. was also established. These results are in agreement with a study conducted by Serrano-Suárez et al. [69], where *Legionella* spp. were isolated when the corresponding heterotrophic plate count concentrations were above 1 × 10^5^ CFU/100 ml, indicating that the frequency at which *Legionella* spp. are isolated may depend on the presence of culturable heterotrophic bacteria. Additionally, positive correlations were established between the heterotrophic plate count and the number of gene copies of viable *Acanthamoeba* spp., *V. vermiformis* and *N. fowleri*. This is expected as these FLAs are heterotrophs and known grazes of bacteria and the heterotrophic plate count represents the general bacterial microbiota [66, 69, 70]. Furthermore, while results indicated that the pH of the unpasteurized and pasteurized tank water samples did not significantly influence the microbiological quality, an increase in temperature of the pasteurized harvested rainwater significantly reduced the heterotrophic plate count, the number of *Legionella* isolates obtained, and the gene copies of viable *Legionella* spp., *Acanthamoeba* spp., *V. vermiformis* and *N. fowleri* detected, respectively.

*Legionella* spp. were also isolated using culture based techniques and during the current study, the majority of the *Legionella* isolates were obtained from unpasteurized tank water samples at temperatures of 18 °C (52 *Legionella* isolates) and 19 °C (15 *Legionella* isolates), respectively. Molecular analysis of the *Legionella* isolates obtained then indicated that *L. longbeachae* was the dominant *Legionella* spp. isolated from the unpasteurized tank water samples. While *L. longbeachae* is generally isolated from soil, including potting soil [71], this microorganism has previously been isolated from water samples collected from hospital reticulation systems and cooling towers and is able to proliferate in *Acanthamoeba polyphaga* [72–74]. In addition, while *L. pneumophila* serotype 1 is responsible for most of the human reported infections, 17 additional species have also been associated with disease and these include *L. longbeachae, L. micdadei*, *L. anisa* and *L. bozemanii* [75]. BLAST analysis also revealed that *L. norrlandica* was of the dominant *Legionella* spp. isolated from unpasteurized tank water samples. This *Legionella* spp. harbours the majority of the *L. pneumophila* virulence factors and has only recently been described, where Rizzardi et al. [76] isolated a novel *Legionella* genus from the biopurification systems of wood processing plants. Moreover, the study group revealed that *L. norrlandica* could establish a replicative vacuole in *A. castellanii*. Three isolates were also identified as *Legionella rowbothamii.* Adeleke et al. [77] reported on the characterization of a novel *Legionella* spp., namely *L. rowbothamii*, and to date no studies have reported on the isolation of *L. rowbothamii* from environmental samples. Moreover, no studies have indicated whether *L. rowbothamii* proliferates in protozoa. As *L. rowbothamii* was isolated during the current study, future research should thus elucidate whether *L. rowbothamii* is able to colonize and proliferate in amoeba species.

Although *Legionella* spp. were isolated from the unpasteurized tank water samples, no *Legionella* spp. were detected using the culture based methods in the pasteurized tank water samples (45 °C to 93 °C). Numerous studies have indicated that temperatures below 50 °C are not sufficient to eradicate *Legionella* spp. from water distribution systems [13, 17, 46, 69] and it is therefore unexpected that *Legionella* spp. were not isolated from particularly the 45 °C pasteurized tank water sample. However, in agreement with previous studies that have focused on the thermal inactivation of *Legionella* spp., the culturability of *Legionella* from the pasteurized tank water samples may have been affected by the heat treatment and by the nutrient shock of going from a nutrient poor environment such as rainwater, onto the nutrient rich environment provided by the media, as this induces *Legionella* cells to enter a viable but non-cultivable (VBNC) state [78, 79].

EMA-qPCR assays were then performed for all unpasteurized and tank water samples pasteurized at 68 °C, 74 °C, 84 °C and 93 °C to determine whether *Legionella* and the FLA’s were viable. Although viable *V. vermiformis* was detected in all the unpasteurized tank water samples, results indicated that solar pasteurization at 74–93 °C was effective in reducing the gene copies of *V. vermiformis* to below the lower limit of detection (< 5–8 gene copies/μl). Additionally, *N. fowleri* were not detected in any of the pasteurized tank water samples and results of the current study thus indicate that the thermal treatment of tank water at 68–93 °C is sufficient for the removal of *N. fowleri* as the gene copies of viable *N. fowleri* were reduced to below the lower limit of detection (< 12–17 gene copies/ μl) for all pasteurized tank water samples*.* Although discrepancies, such as the presence of multicellular communities, may arise when analysing environmental samples, Fouque et al. [31] indicated that *V. vermiformis* cysts were completely inactivated at 70 °C, which is in agreement with the current study, where *V. vermiformis* was more sensitive to heat treatment than *Acanthamoeba* spp. Additionally, previous studies have indicated that *Naegleria* spp. are considered more sensitive to heat treatments compared to thermotolerant *Acanthamoeba* and *V. vermiformis* [24, 80, 81]. Results obtained in the current study do however, indicate that rainwater harvesting tanks are vulnerable to *N. fowleri* and *V. vermiformis* colonization and amoeba, including *N. fowleri* and *V. vermiformis,* should be included in the surveillance of pathogens in drinking water distribution systems [82].

Viable *Legionella* spp. and *Acanthamoeba* spp. were then detected in all the pasteurized and unpasteurized tank water samples. It has been well established that Legionellae are facultative intracellular parasites of amoeba including, *Acanthamoeba* spp., *Naegleria* spp. and *V. vermiformis* [83]. Although significant positive correlations were observed between the number of *Legionella* gene copies and the gene copies of *V. vermiformis* and *N. fowleri*, it is hypothesized that *Legionella* spp. may primarily be associating with *Acanthamoeba* spp. during thermal treatment as viable *Legionella* spp. and *Acanthamoeba* spp. persisted in all pasteurized (68 °C to 93 °C) and unpasteurized tank water samples. This is not surprising, as previous studies have detected *Legionella* at high pasteurization temperatures (> 90 °C) in solar pasteurization systems using molecular based techniques, including EMA-qPCR [13, 17]. Although this has not been demonstrated for all *Legionella* spp., *L. pneumophila* has been known to survive and proliferate on the debris of dead microbial cells such as heat-killed *Pseudomonas putida*, *E. coli*, *Bacillus subtilis*, *Lactobacillus plantarum*, *A. castellanii* and *Saccharomyces boulardii* [84]. Moreover, *Acanthamoeba* spp. are able to graze on heat killed bacteria including *E. coli* and *Klebsiella* spp. [70]. In agreement with Thomas et al. [81], it is thus hypothesized that the solar pasteurization system may be indirectly providing favourable conditions for *Legionella* and *Acanthamoeba* spp. and these organisms may thus be surviving on the dissolved organic constituents, available through the decay of the microorganisms at high pasteurization temperatures. However, while the presence of dissolved organic constituents may allow for the survival of *Legionella* spp., it is hypothesized that *Acanthamoeba* cysts may be harbouring *Legionella* and allow the *Legionella* spp. to proliferate and grow in harvested rainwater and during the treatment process. This is in agreement with Storey et al. [85] who indicated that Acanthamoebae cysts remained viable after heat treatment at 80 °C for 10 min. It is further hypothesized that during the DNA extraction process the cysts may be lysed and *Legionella* are released and detected using molecular methods including EMA-qPCR. Additionally, the qPCR assays utilized in the current study indicated high reproducibility as the mean inter-assay CV values and SD were less than 5 and 1 %, respectively, which were comparable to CV and SD values obtained by Ahmed et al. [65]. The current study therefore highlights the value of EMA-qPCR for the detection of viable *Legionella* and their protozoan hosts as opposed to culture based techniques that may yield false negative results [82].

## Conclusions

Although incidences of Legionnaires’ disease are well documented for regions including Europe, the USA, New Zealand and Australia, limited information is available on the environmental distribution of *Legionella* spp. as well as incidences of Legionnaires’ disease in developing countries such as South Africa [83, 86–88]. The surveillance of *Legionella* in water distribution systems is thus vital as *Legionella* have been described as “new or emerging pathogens in drinking water” [89]. The current study demonstrated that culture-based methods for the detection of *Legionella* are less sensitive and with the use of EMA-qPCR, viable *Legionella* spp.*, Acanthamoeba* spp.*, V. vermiformis* and *N. fowleri* were detected in untreated tank water samples, while viable *Legionella* spp. (93 °C)*, Acanthamoeba* spp. (93 °C) and *V. vermiformis* (68 °C) were detected after pasteurization.

Additionally, insight into the presence and persistence of *Legionella* spp., and amoeba including *Acanthamoeba*, *V. vermiformis* and *N. fowleri* in a representative rainwater harvesting tank and a solar pasteurization treatment system was provided. The occurrence of these pathogens in harvested rainwater is of particular concern as they are frequently detected in water distribution systems and residential plumbing [45]. For example, *N. fowleri* is the causative agent of the disease, primary amoebic meningoencephalitis (PAM) and although PAM infections are rare, the mortality rate is extremely high [90].

The presence of viable *Legionella* spp. and *Acanthamoeba* spp. highlights the need for further investigation as solar pasteurization may be insufficient for the long-term control of pathogenic *Acanthamoeba* and Acanthamoebae-bound Legionellae in harvested rainwater.

## Abbreviations

ACES: *N*-(2-acetamido)-2-aminoethanesulfonic acid
BCYE: Buffered charcoal yeast extract
BLAST: Basic local alignment search tool
BYE: Buffered yeast extract
CAF: Central analytical facility
CV: Coefficient of variation
CYE: Charcoal yeast extract
Dot/Icm: Defect in organelle trafficking/intracellular multiplication
DWAF: Department of Water Affairs and Forestry
EMA: Ethidium monoazide
EMA-qPCR: Ethidium monoazide quantitative polymerase chain reaction
EPF: End-point fluorescence
FLA: Free-living amoeba
gDNA: Genomic DNA
GHI: Global horizontal irradiance
GVPC: Glycine, vancomycin, polymyxin B and cycloheximide
NA: Nutrient agar
NCBI: National centre for biotechnology information
qPCR: Quantitative PCR
R2A: Reasoner’s 2A agar
RWH: Rainwater harvesting
SD: Standard deviation
VBNC: Viable but non-culturable
